# Navy, Army, Air and Colonial Services

**Published:** 1921-08-27

**Authors:** 


### NAVY, ARMY, AIR AND COLONIAL SERVICES.

### ROYAL NAVAL MEDICAL SERVICE.

Direct entry to the Permanent Service is for the present in abeyance. The Regulations published in The Hospital of September 4, 1920, page 587, stand with the following addition and alterations to clause (5) that candidates who have served during the war in the Royal Navy, Army, or Air Force, either as officers or men, but who have not been members of the Officers' Training Corps, will be accorded 3 per cent. additional marks in the Entrance examination if their services are under one year, and 6 per cent. if over that period. Candidates who have served in the Officers' Training Corps, who are in possession of certificate A, receive 3 per cent., and those in possession of certificates A and B 6 per cent. of the maximum number of marks allotted.

*Surgeon-Lieutenants for Short Service.*—The following are the qualifications and conditions for the entry of duly qualified men as surgeon-lieutenants for short periods: (1) Age not to exceed thirty; (2) recommendation by the Deans of their schools; (3) certificate of good character; (4) unmarried candidates preferred. Pay 26s. 6d. a day, or £483 12s. 6d. yearly. *Gratuity* on discharge, £8 6s. 8d. for each complete month of service. Period of engagement three years, with option to continue for twelve months further if required. After six months' service a short-service surgeon-lieutenant who was less than twenty-eight years when joining, may, if desired, be considered for transfer to the Permanent Service.

### ROYAL ARMY MEDICAL CORPS.

A candidate for a commission in the Royal Army Medical Corps must fulfil certain physical and medical requirements, and must be 21 years and not over 28 years of age at the date of the commencement of the Entrance examination. He must be registered under the Medical Acts in force in the United Kingdom. The Entrance examination is held twice a year in London—January and July—a fee of £1 being charged for admission thereto. It lasts about three days. The examination is of a clinical, written, practical, and oral character. A candidate who is successful at the Entrance examination is appointed a lieutenant (on probation), and is then required to attend at the Royal Army Medical College, London, and afterwards at the Royal Army Medical Corps School of Instruction, for the purpose of qualifying in certain subjects before his commission can be confirmed.

The scale of pay of officers of the Royal Army Medical Corps varies according to length of service, and, including all allowances, is summarised as follows: Lieutenant, married £617, unmarried £550; captain, married £672 to £836, unmarried £668 to £741; major, married £909 to £1,000, unmarried £847 to £939; lieutenant-colonel, married £1,193 to £1,274, unmarried £1,130 to £1,221; colonel, married £1,502, unmarried £1,433; major-general, married, £2,212, unmarried £2,139. Many officers receive charge pay and specialists' pay in addition. Retired pay: *Service element*, £15 per year for each completed year of service; *Rank element* maximum, major £120; lieutenant-colonel £240. There is no rank element authorised for ranks lower than major.

### ROYAL AIR FORCE MEDICAL SERVICE.

(*A description of this Service appears on p. 360.*)

A candidate must be of pure European descent and the son of a subject of the British Empire. He must be under twenty-eight years of age, and be nominated by the Dean of a recognised medical school. An outfit allowance of £50 will be granted to officers if they have had no previous commissioned service. In the latter case they receive an outfit allowance of £25. The rates of pay rise from 24s. a day on joining as flying officer to 60s. a day on promotion to group captain, which increases to 68s. a day after six years in the substantive rank. For service in India the pay rises from 650 rupees per mensem as flying officer to 1,950 rupees as principal medical officer. The maximum retired pay for group captains retiring at fifty-five is £900; for wing commanders retiring at fifty-one, £600; for squadron leaders retiring at forty-eight, £500. The minimum qualifying period for retired pay is twenty years. Short-service officers receive gratuities at the rate of £100 for each of the first two completed years of service, and at the rate of £150 for the third and fourth completed year. If granted permanent commissions these gratuities will not be payable, but the period of service under the short-service commission will count for retired pay. Medical officers holding permanent commissions may at the discretion of the Air Council be allowed to withdraw from the Service after ten years' service with a gratuity of £1,250, or after sixteen years' with a gratuity of £2,000 in lieu of retired pay. The usual allowances will be payable to medical officers under the same conditions as to officers of other branches of the Royal Air Force.

### INDIAN MEDICAL SERVICE.

The open competitive examination has been in suspense since July 1915, and the present procedure for appointment to the Indian Medical Service is by nomination by the Secretary of State for India on the recommendation of a Selection Committee. Applications should be addressed to the Secretary, Military Department, India Office, Whitehall, London, S.W. 1, and should contain concise particulars of the applicant's medical degrees and career. Applicants must be over twenty-one and under thirty-two years of age at the time of application. Particulars regarding pay, promotion, etc., in the Service can be obtained from the Military Department, India Office. Every candidate must be a British subject of European or East Indian descent, and his father must at the time of the candidate's birth have been either a British subject born within His Majesty's allegiance, or a person to whom a certificate of naturalisation had been granted, or a subject of a State in India under the suzerainty of His Majesty. Every candidate must also be of sound bodily health, and, in the opinion of the Secretary of State for India in Council, in all respects suitable to hold a commission in the Indian Medical Service. He may be married or unmarried. He must possess a qualification registrable in Great Britain and Ireland under the Medical Acts in force at the time of his appointment. The physical fitness of each candidate will be determined by a Board of Medical Officers, who are required to certify that his vision is sufficiently good to enable him to pass the tests laid down by the regulations. The rates of pay are according to length of service. They may be summarised as follows:—Total basic pay and overseas allowances per mensem: Lieutenant, 650 rupees; captain, 800 to 1,050 rupees; major, 1,200 to 1,500 rupees; lieutenant-colonel, 1,750 to 2,100 rupees. Charge allowances and second-in-command allowances for station hospitals are extra. There are some special provisions in respect of the pay of Indian officers.

### COLONIAL MEDICAL APPOINTMENTS.

Applicants should be between the ages of twenty-three and thirty-five (thirty-seven in the case of the Straits Settlements and the Malay States). Candidates must be doubly qualified, and those who have previously held hospital appointments are preferred. Before appointment they will have to submit to medical examination by one of the consulting physicians of the Colonial Office.

In the majority of instances the duties required of a Colonial medical officer are of a general character. They include medical, surgical, and not infrequently public health work. Occasionally only is a specialist required.

Vacancies at present exist in East Africa, in the West African Medical Staff, in the Straits Settlements and Federated Malay States, in Fiji, and certain of the West Indian Colonies. In a number of cases salaries have recently been improved. There are a few vacancies for health officers. Full details may be obtained on application to the Assistant Private Secretary (Appointments), Colonial Office, S.W.

### ARMY DENTAL CORPS.

A limited number of permanent commissions will be given to persons not over forty years of age who are duly qualified under regulations approved by the Army Council. Appointments will for the present be limited to candidates who are now serving or who have served as dental officers during the war. An officer will be eligible for promotion to the rank of captain on completion of three and a-half years' service in the Corps, provided he has previously qualified in such manner as may be prescribed by the Army Council. Appointments will be in the rank of lieutenant except in cases where service of three and a-half years or more has been given as a temporary dental officer, when the rank of captain will be given. Service as a temporary dental officer will count towards the three and a-half years for promotion to captain. The pay and allowances amount to £553 per annum to a married lieutenant, or £495 if unmarried, rising by scale through the various ranks to £1,096 on promotion to be lieutenant-colonel (married) and £1,052 if unmarried. In this rank after twenty-five years' service the pay and allowances are £1,187 (married), £1,144 (unmarried). The rates of retired pay are the same as for officers of the Royal Army Medical Corps (which see). Voluntary retirement on retired pay will not be allowed until after twenty years' service. Earlier retirement on gratuity may be allowed after eight and a-half years' service in the Corps, gratuity of £1,000; after fifteen years' service in the Corps, gratuity of £1,800; after eighteen years' service in the Corps, gratuity of £2,500. Retirement is compulsory at the age of fifty-five years. Intending candidates should make application for the necessary forms to the Secretary, War Office (A.M.D.1), Whitehall, S.W. Officers who are now serving should submit their applications through the usual official channels.

